# Improved Particle Swarm Optimization Algorithm Based on Last-Eliminated Principle and Enhanced Information Sharing

**DOI:** 10.1155/2018/5025672

**Published:** 2018-12-05

**Authors:** Xueying Lv, Yitian Wang, Junyi Deng, Guanyu Zhang, Liu Zhang

**Affiliations:** ^1^College of Instrumentation & Electrical Engineering, Jilin University, Changchun 130061, China; ^2^College of Computer Science and Technology, Jilin University, Changchun 130022, China; ^3^National Engineering Research Center of Geophysics Exploration Instruments, Jilin University, Changchun 130061, China

## Abstract

In this study, an improved eliminate particle swarm optimization (IEPSO) is proposed on the basis of the last-eliminated principle to solve optimization problems in engineering design. During optimization, the IEPSO enhances information communication among populations and maintains population diversity to overcome the limitations of classical optimization algorithms in solving multiparameter, strong coupling, and nonlinear engineering optimization problems. These limitations include advanced convergence and the tendency to easily fall into local optimization. The parameters involved in the imported “local-global information sharing” term are analyzed, and the principle of parameter selection for performance is determined. The performances of the IEPSO and classical optimization algorithms are then tested by using multiple sets of classical functions to verify the global search performance of the IEPSO. The simulation test results and those of the improved classical optimization algorithms are compared and analyzed to verify the advanced performance of the IEPSO algorithm.

## 1. Introduction

The development of industrial society has led to the successful application of the optimal design methods to diverse engineering practices, such as path planning, structural design, control theory, and control engineering [1–10]. In 1995, the foraging behavior of bird swarm inspired Kennedy and Eberhart to propose the particle swarm optimization (PSO) algorithm. PSO requires few parameter adjustments and is easy to implement; hence, it is the most commonly used swarm intelligence algorithm [11–20]. However, in practical applications, most problems are complicated design problems with multiple parameters, strong coupling, and nonlinearity. Therefore, improving the global optimization capability of an optimization algorithm is important in solving complex engineering optimization problems. To improve the capability of traditional PSO, many scholars have proposed improvement strategies, including the adjustment of parameters and combinations of various mechanisms.

Shi and Eberhant [21] proposed an inertial weight improvement strategy (SPSO) with strong global search capability at the beginning of an iteration, strong local search capability in the latter iteration, and fine search near the optimal solution. Although the SPSO improves the convergence speed of the algorithm, the “premature” phenomenon remains. Zhang [22] proposed an improved PSO algorithm with adaptive inertial weight that is based on Bayesian technology to balance the development and exploration capability of populations. Ratnawecra [23] proposed a linear adjustment method for learning factors. In the early stages of the iteration, the particle flight was mainly based on the historical information of the particle itself, and the latter particle flight was mainly based on the social information between the particle and the global optimal particle. However, this method still has defects. The best fit for the initial global search is similar to the local optimum. Moreover, convergence is only limited to some optimal regions rather than globally, thereby causing the PSO algorithm to fall into the local extrema. Chen and Ke [24] proposed a chaotic dynamic weight (CDW) PSO (CDW-PSO) algorithm. Chaotic maps and dynamic weights were used to modify the search process. Although CDW-PSO indicates an improved search performance relative to other natural heuristic optimization algorithms, it also easily falls into the local optimum. Chen [25] proposed a dynamic multiswarm differential learning PSO (DMSDL-PSO) algorithm, in which the differential evolution method is applied to each subgroup combined with a differential mutation method to conduct a global search, and a quasi-Newton method is applied for local search. The DMSDL-PSO algorithm has good exploration and exploitation capabilities. Jiang [26] proposed a new binary hybrid PSO with wavelet mutation (HPSOWM), in which the motion mechanism and mutation process of particles are converted into binary elements and the problem is transformed from a continuous space problem into a discrete domain one. Although the convergence speed of the HPSOWM algorithm is stable and robust, its convergence rate is lower than those of other intelligent optimization algorithms. To solve the dynamic multiobjective optimization problem with rapid environmental change, a study proposed a cooperative multiswarm PSO for dynamic multiobjective optimization (CMPSODMO) [27]. In comparison with other dynamic multiobjective optimization algorithms, CMPSODMO indicates a better effect in addressing uncertain rapid environmental changes. Ye [28] proposed a multiswarm PSO algorithm with dynamic learning strategies, in which the population is divided into ordinary and communication particles. The dynamic communication information of communication particles was applied to the algorithm to maintain particle population diversity. Although this method improves the capability of the algorithm to handle complex multimodal functions, it increases the computational complexity of the algorithm. Cui [29] proposed a globally optimal prediction-based adaptive mutation PSO (GPAM-PSO) to avoid the local optimal problem of traditional PSO algorithms. However, GPAM-PSO is limited to the dimensionality reduction of nonzero mean data. Zhang [30] proposed a vector covariance PSO algorithm that divides all the dimensions of a particle into several parts randomly and optimizes each part to enhance the global and local search capabilities. However, the algorithm continues to fall into the local extrema.

PSO has attracted considerable research attention due to its easy implementation, few parameter adjustments, and adaptability. Scholars use PSO to solve engineering optimization problems and gradually penetrate various fields of application, such as parameter optimization, path planning, predictive control, and global optimization. Zhao [31] used PSO to optimize wavelet neural network parameters, reduce the limitations of the assessment of network security situational awareness, and thereby meet the requirements of network security in a big data environment. The parameter-related coefficients in a nonlinear regression analysis model were optimized by combining particle swarm with a genetic phase [32] to reduce the vibrations caused by mine blasting that damages the structures around the blasting area. The derived diffusion-free PSO algorithm was used to estimate the parameters of an infinite impulse response system and improve the energy utilization of an infinite sensor network [33]. Wang [34] used a multiobjective PSO algorithm to solve a path-planning problem of mobile robots in a static rough terrain environment. Wang [35] combined PSO with chaos optimization theory to establish a mathematical model of a path-planning problem in the radioactive environment of nuclear facilities to ensure personnel safety. Lopes [36] proposed a novel particle swarm-based heuristic technique to allocate electrical loads in an industrial setting throughout the day. Multiobjective PSO was used to solve the problem of service allocation in cloud computing [37]. Petrovic [38] proposed chaos PSO to achieve an optimal process dispatching plan. Zhang [39] proposed an adaptive PSO to solve problems in reservoir operation optimization with complex and dynamic nonlinear constraints.

An improved PSO algorithm was used for a time-series prediction of a grayscale model [40]. The algorithm reduces the average relative error between the recovery and measured values of the model to avoid the problems caused by the optimization of background values. Gulcu [41] used PSO to establish a power demand forecasting model.

In view of the aforementioned methods, an improved PSO (IEPSO) algorithm is proposed in the present work. In IEPSO, the last-eliminated principle is used to update the population and maintain particle population diversity. The global search capability of the IEPSO algorithm is improved by adding local-global information sharing terms. A multigroup test function is used for comparison with the IEPSO. A classical optimization algorithm and its improved versions are used to test and verify the global optimization performance of the IEPSO algorithm.

## 2. IEPSO

### 2.1. Standard PSO

The initial population of the PSO algorithm is randomized. The IEPSO updates the position and speed of the particle swarm by adaptive learning, as shown in following formulas:

(1) $$\begin{array}{c} v_{id}^{t+1}=\omega v_{id}^{t}+C_{1}R_{1}\left(p_{id}^{t}-x_{id}^{t}\right)+C_{2}R_{2}\left(p_{gd}^{t}-x_{id}^{t}\right),\\ x_{id}^{t}=x_{id}^{t}+v_{id}^{t+1}, \end{array}$$

where *ω* is the inertial weight, *C*_1_ and *C*_2_ are the acceleration terms, *R*_1_ and *R*_2_ are the random variables uniformly distributed in the range of (0, 1), *P*_*g*_^*t*^ is the global better position, *P*_*i*_^*t*^ is the particle that finds the best position in history, *x*_*i*_^*d*^ is the particle position in the current iteration, and v_*id*_^*t*+1^ is the particle update speed at the next iteration.

### 2.2. IEPSO

**Figure 1:**
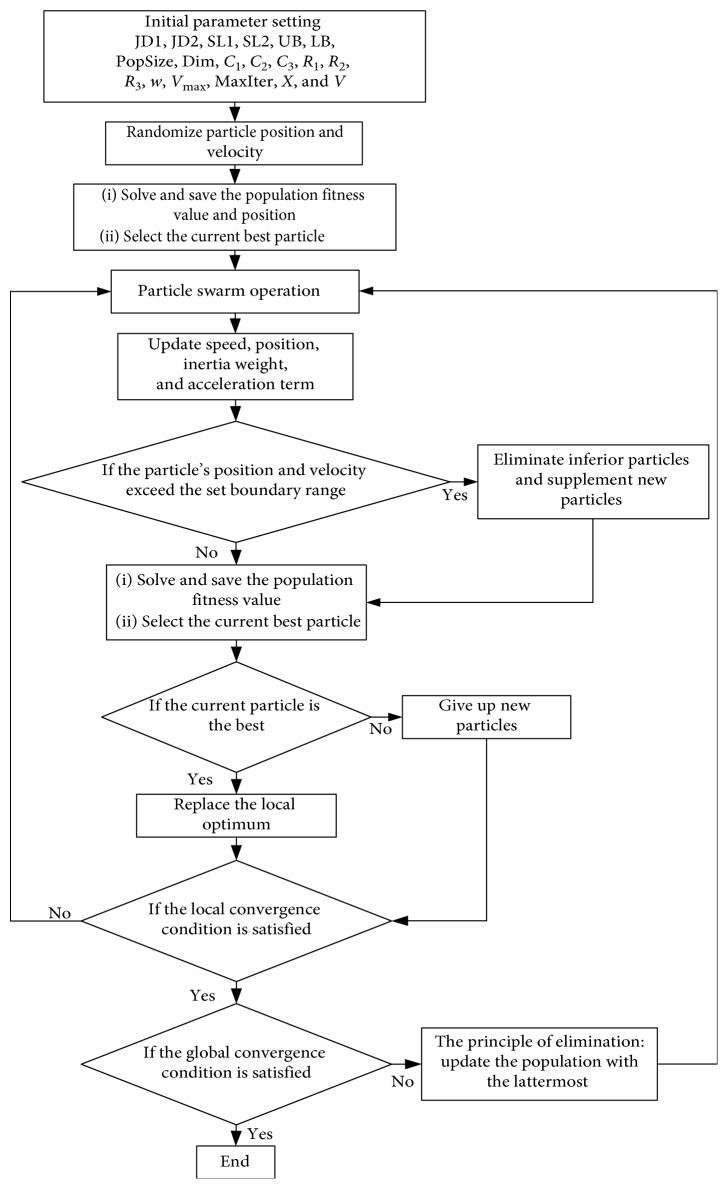
IEPSO algorithm flowcharts.

The IEPSO algorithm is mainly based on the last-eliminated principle and enhances the local-global information sharing capability to improve its global optimization performance. The specific implementation of the IEPSO algorithm is shown in Figure 1.

The position and velocity of particles in a population are randomly initialized, and the fitness value of the particles is calculated. Information on the current individual and global optimal particles, including their positions and fitness values, is saved. Then, the particle swarm operation is conducted. In the IEPSO algorithm, Formula (2) is used to update the speed to balance the exploration and exploitation capabilities of the particles in the global optimization process. Formula (3) is the local-global information sharing term:

(2) $$\begin{array}{c} v_{id}^{t+1}=\omega v_{id}^{t}+C_{1}R_{1}\left(p_{id}^{t}-x_{id}^{t}\right)+C_{2}R_{2}\left(p_{gd}^{t}-x_{id}^{t}\right)+C_{3}R_{3}\left|p_{gd}^{t}-p_{id}^{t}\right|, \end{array}$$

(3) $$\begin{array}{c} \varphi_{3}=C_{3}R_{3}\left|p_{gd}^{t}-p_{id}^{t}\right|. \end{array}$$

Formula (2) comprises four parts, namely, the inheritance of the previous speed, particle self-cognition, local information sharing, and “local-global information sharing.”

The IEPSO algorithm is not limited to one-way communication between global and individual particles. The local-global information sharing term (*φ*_3_) is added to the information exchange between the local optimum and global optimal particles obtained by the current iteration, and the population velocity is updated by Formula (2). In the early stage of the algorithm, the entire search space is searched at a relatively high speed to determine the approximate range of the optimal solution; the result is beneficial for global search. In the latter stage, most of the particle search space is gradually reduced and concentrated in the neighborhood of the optimal value for deep search; the result is beneficial for local search.

The particles that have not exceeded the predetermined range after the speed update continue to retain their original speed. The maximum value of the velocity is assigned to the particle that is beyond the predetermined range after the speed is updated. The particles that have not exceeded the predetermined range after the location update continue to retain their original positions. When the particles are beyond the predetermined range, inferior particles are eliminated by adding new particles to the population within the predetermined range, thereby forming a new population. The fitness value of the new population is recalculated, and the information of the individual particle and its global optimal position and fitness value obtained by the current iteration are preserved. In all the algorithms, particles have good global search capability at the beginning of the iteration, and as individual particles move closer to the local optimal particle, the algorithms gradually lose particle diversity. On the basis of the idea of population variation of the traditional genetic algorithm (GA), the last-eliminated principle is applied in the IEPSO algorithm to maintain particle population diversity. When the PSO satisfies the local convergence condition, the optimal value obtained at this time may be the local optimal value. Particle population diversity is maintained by using the particle fitness function as the evaluation criterion, thereby eliminating particles with poor fitness or high similarity. New particles are added to a new species in a predetermined range, and the particle swarm operations are reexecuted. If the number of the current iteration reaches the required predefined convergence accuracy, the iteration is stopped, and the optimal solution is produced. The complexity and runtime of the algorithm increase due to the increased local-global information sharing and the last-eliminated principle. Nevertheless, experimental results show that the improved method can enhance the accuracy of the algorithm.

## 3. Experimental Study

Eleven test functions are adopted in this study to test the performance of the proposed IEPSO. In this test, *f*_1_–*f*_5_ are unimodal functions, whereas *f*_6_–*f*_11_ are multimodal functions. *f*_6_ (Griewank) is a multimodal function with multiple local extrema, in which achieving the theoretically global optimum is difficult. *f*_7_ (Rastrigin) possesses several local minima, in which finding the global optimal value is difficult. *f*_10_ (Ackley) is an almost flat area modulated by a cosine wave to form a hole or a peak; the surface is uneven, and entry to a local optimum during optimization is easy. *f*_11_ (Cmfun) possesses multiple local extrema around the global extremum point, and falling into the local optimum is easy.  Table 1 presents the 11 test functions, where *D* is the space dimension, *S* is the search range, and CF is the theoretically optimal value.

### 3.1. Parameter Influence Analysis of Local-Global Information Sharing Term

This study proposes the addition of a local-global information sharing term, which involves the parameter *C*_3_. Therefore, the following exploration is conducted in a manner in which *C*_3_ is selected by using the 11 test functions.

1. When *C*_3_ takes a constant value, constant 2 is selected.
2. The linear variation formula of *C*_3_ is as follows:

(4) $$\begin{array}{c} C_{3}=k\left(C_{3}\_start-\left(C_{3}\_\text{start}-C_{3}\_\text{end}\right)\times \frac{t}{t_{\max}}\right), \end{array}$$

where *k* is the control factor. When *k* = 1, *C*_3_ is a linearly decreasing function; when *k* = −1, *C*_3_ is a linearly increasing function. *C*_3__start and *C*_3__end are the initial and termination values of *C*_3_, respectively. *T* is the iteration number, and *t*_max_ is the maximum number of iterations.

**Figure 2:**
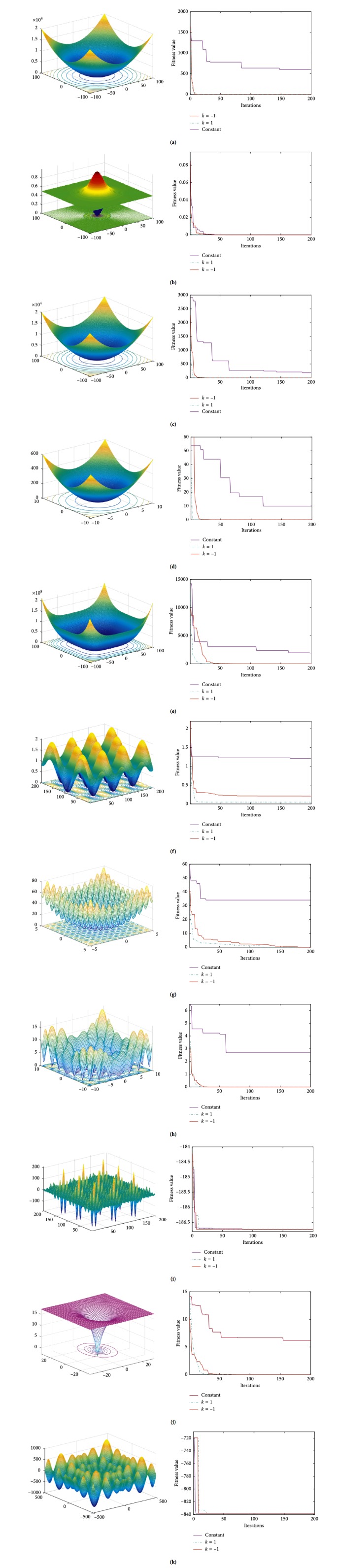
11 test functions: (a) *f*_1_ sphere function; (b) *f*_2_ Schaffer function; (c) *f*_3_ step function; (d) *f*_4_ SumSquares function; (e) *f*_5_ Zakharov function; (f) *f*_6_ Griewank function; (g) *f*_7_ Rastrigin function; (h) *f*_8_ alpine function; (i) *f*_9_ Shubert function; (j) *f*_10_ Ackley function; (k) *f*_11_ Cmfun function.

Tables 2 and 3 and Figure 2 show that *C*_3_ is a constant that linearly declines and linearly increases in three cases. When the parameter *C*_3_ of the local-global information sharing term is a linearly decreasing function, the average fitness value of the testing function is optimal, and the convergence speed and capability to jump out of the local extrema are higher than those in the other two cases. When *C*_3_ takes a constant, the algorithm cannot balance the global and local search, resulting in a “premature” phenomenon. When *C*_3_ adopts the linearly decreasing form, the entire area can be quickly searched at an early stage, and close attention is paid to local search in the latter part of the iteration to enhance the deep search ability of the algorithm. While *C*_3_ adopts a linearly increasing form, it focuses on the global-local information exchange in the latter stage of the iteration. Although this condition can increase the deep search ability of the algorithm, it will cause the convergence speed to stagnate. Therefore, compared with the linearly increasing form, the linearly decreasing form shows a simulation curve that converges faster and with higher precision.

Therefore, the selection rules of the parameter *C*_3_ of local-global information sharing in a decreasing function are investigated in this study. The nonlinear variation formula of *C*_3_ is as follows:

(5) $$\begin{array}{c} C_{3}=\left(C_{3}\_\text{start}-C_{3}\_\text{end}\right)\times \tan\left(0.875\times {\left(1-\frac{t}{t_{\max}}\right)}^{k}\right)+C_{3}\_\text{end,} \end{array}$$

where *C*_3__start and *C*_3__end are the initial and termination values of the acceleration term *C*_3_, respectively, and *k* is the control factor. When *k* = 0.2, *C*_3_ is a convex decreasing function; when *k* = 2, *C*_3_ is a concave decreasing function. *t* is the iteration number, and *t*_max_ is the maximum number of iterations.

Table 4 shows that when *C*_3_ is a convex function, the precision and robustness of the algorithm can obtain satisfactory results on *f*_1_–*f*_5_.  Table 5 shows that when *C*_3_ is a convex function, the algorithm obtains a satisfactory solution and shows a fast convergence rate on *f*_6_, *f*_8_, *f*_9_, *f*_10_, and *f*_11_. In the unimodal test function, the IEPSO algorithm does not show its advantages because of its strong deep search capability. In the complex multimodal test function, when the convex function is used in *C*_3_, the downward trend is slow in the early stage, thus benefiting the global search, and the downward speed increases in the later stage, thus benefiting the local search. When the concave function is used for *C*_3_, the descent speed is fast in the early stage. Although the search speed is improved, the coverage area of the search is reduced, thereby leading to the convergence of the algorithm to the nonoptimal value. From the simulation diagrams (f)–(*k*), the convergence speed is observed to be slightly slow when *C*_3_ is a convex function, but its ability to jump out of the local extremum and the accuracy of the global search are higher than those in the other two cases. When *C*_3_ is a concave function, the convergence speed is faster than those in the other two cases, and the search accuracy is lower than that when *C*_3_ is a convex function.

### 3.2. Comparison of Test Results

**Figure 3:**
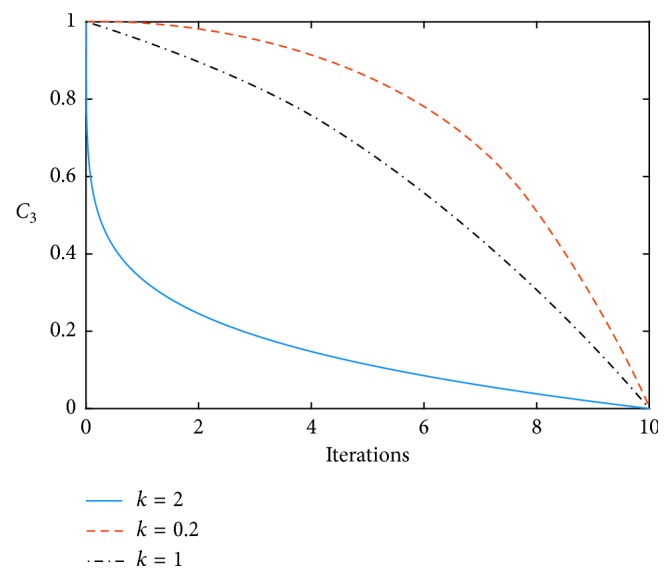
The change curve of *C*_3_ with the number of iterations.

**Figure 4:**
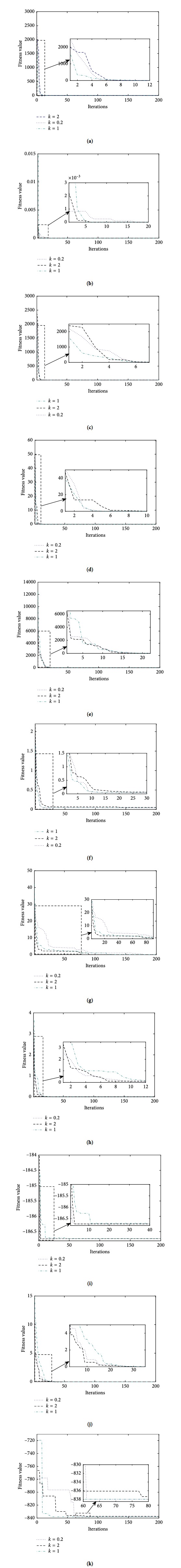
11 test functions: (a) *f*_1_ sphere function; (b) *f*_2_ Schaffer function; (c) *f*_3_ step function; (d) *f*_4_ SumSquare function; (e) *f*_5_ Zakharov function; (f) *f*_6_ Griewank function; (g) *f*_7_ Rastrigin function; (h) *f*_8_ alpine function; (i) *f*_9_ Shubert function; (j) *f*_10_ Ackley function; (k) *f*_11_ Cmfun function.

The 11 test functions in Figure 1 are used to compare the IEPSO algorithm with classical PSO, SPSO, differential algorithm (DE), and GA. The DE, GA, and PSO algorithms are all stochastic intelligent optimization algorithms with population iterations. The evaluation criteria of algorithm performance include speed of convergence and size of individual population search coverage. The differential optimization algorithm has a low space complexity and obvious advantages in dealing with large-scale and complex optimization problems. The GA has good convergence when solving discrete, multipeak, and noise-containing optimization problems. Based on the traditional PSO algorithm, the SPSO algorithm achieves the balance between global search and local search by adjusting the inertial weight (Figures 3 and 4).

**Figure 5:**
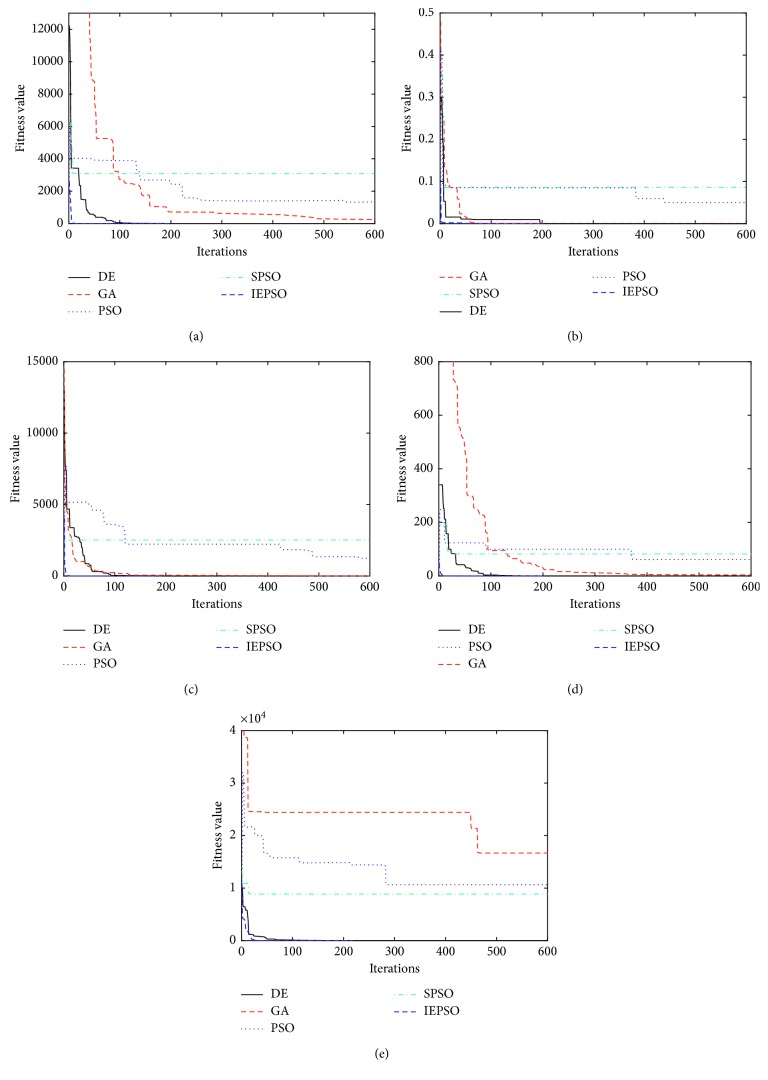
Unimodal functions: (a) *f*_1_ sphere function; (b) *f*_2_ Schaffer function; (c) *f*_3_ step function; (d) *f*_4_ SumSquares function; (e) *f*_5_ Zakharov function.

**Figure 6:**
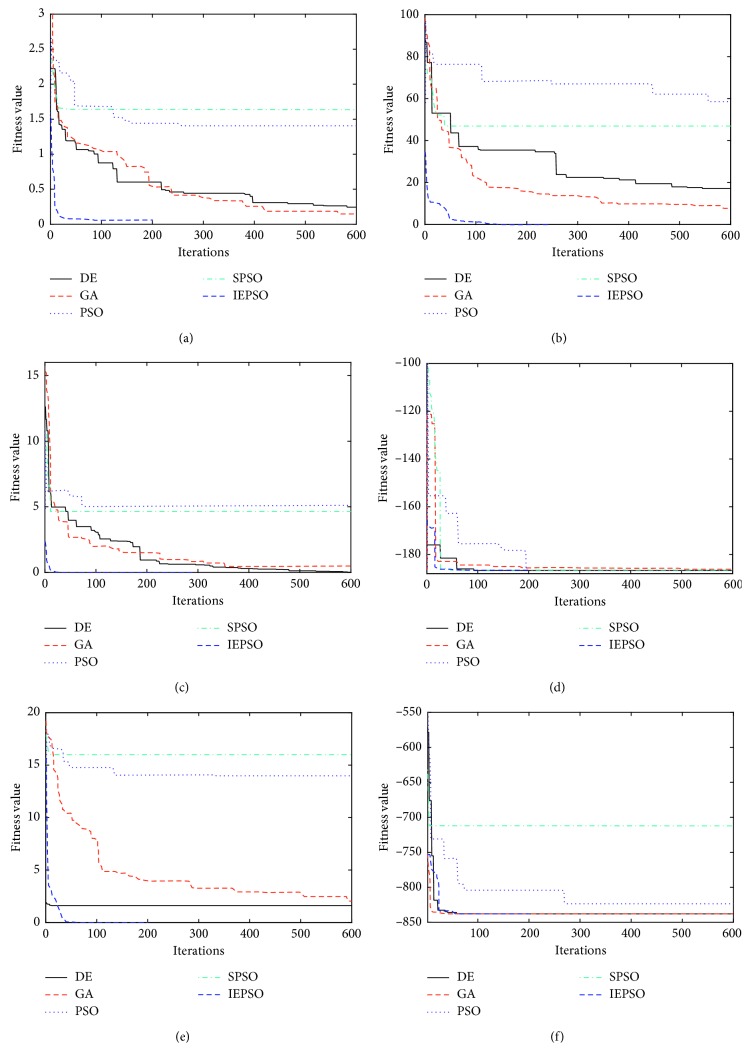
Multimodal functions: (a) *f*_6_ Griewank function; (b) *f*_7_ Rastrigin function; (c) *f*_8_ alpine function; (d) *f*_9_ Shubert function; (e) *f*_10_ Ackley function; (f) *f*_11_ Cmfun function.

The experimental parameters of the five algorithms are set, as shown in Table 6. Each test function is run independently 10 times, and the average is recorded to reduce the data error. The iteration is stopped when the convergence condition meets the convergence accuracy. The best average fitness value of the five algorithms is blackened. The standard deviation, average fitness, and optimal value of each algorithm are shown in Tables 7 and 8; Figures 5 and 6 plot the convergence curves of the 11 test functions.

Table 7 shows that the IEPSO has the best performance on *f*_1_, *f*_2_, *f*_3_, and *f*_4_. The IEPSO algorithm obtains the theoretical optimal value on *f*_2_. DE can search the global solution on *f*_5_. The deep search capability of the IEPSO algorithm is considerably higher than that of the PSO and SPSO algorithms due to the increased global-local information sharing term and the last-eliminated principle. The crossover, mutation, and selection mechanisms make the DE algorithm perform well in the early stage of the global search. However, the diversity of the population declines in the latter stage because of population differences. The simulation diagrams (a)–(e) show that although the DE algorithm converges rapidly in the early stage, its global search performance in the later stage becomes lower than that of the IEPSO algorithm. When the GA is used to solve optimization problems, the individuals in the population fall into the local optimum and do not continue searching for the optimum solution. Therefore, in Figure 5, the simulation curve of the GA converges to the local optimum.

The test results in Table 8 indicate that the IEPSO has the best performance on *f*_6_, *f*_7_, *f*_8_, *f*_9_, *f*_10_, and *f*_11_ and that the DE and GA can obtain the theoretical optimal value on *f*_9_ and *f*_11_. Although the GA and IEPSO algorithm can obtain the global optimal value on *f*_9_, the IEPSO algorithm is more robust than the GA is. As shown in the simulation curve of Figure 6, the diversity of the population is maintained because the supplementary particles in the population are stochastic when the local optimal solution converges gradually. The IEPSO algorithm can jump out of the local extrema points in the face of complex multimodal test functions, and the number of iterations required is correspondingly reduced.

Table 9 shows the test results for the three improved PSO algorithms. The DMSDL-PSO algorithm in [25] is a PSO algorithm combined with differential variation and the quasi-Newton method, whereas the HPSOWM algorithm in [26] is a binary PSO algorithm based on wavelet transform. Table 9 shows that the IEPSO algorithm obtains the best value in 5 out of the 11 test functions, and the above analysis indicates that the IEPSO outperforms the other improved PSO algorithms.

## 4. Conclusion

In contemporary engineering design, solving the global optimization problems of multiparameter, strongly coupled, and nonlinear systems using conventional optimization algorithms is difficult. In this study, an improved PSO, that is, the IEPSO algorithm, is proposed on the basis of the last-eliminated principle and an enhanced local-global information sharing capability. The comparison and analysis of the simulation results indicate the following conclusions:

1. The exchange of information between global and local optimal particles enhances the deep search capability of the IEPSO algorithm.
2. The standard test function is used to simulate the parameter *C*_3_ of the local-global information sharing term. The results show that the global optimization capability of the IEPSO algorithm is strong when *C*_3_ is linearly decreasing. Moreover, the proposed algorithm can show the best search performance when *C*_3_ is a nonlinear convex function.
3. The last-eliminated principle is used in the IEPSO to maintain particle population diversity. Moreover, PSO is avoided in the local optimal value. A comparison of the IEPSO algorithm with the classical optimization algorithm and its improved versions verifies the global search capability of the IEPSO algorithm.

In summary, the comparative results of the simulation analysis reveal that, with the application of the last-eliminated principle and the local-global information sharing term to the IEPSO, the proposed algorithm effectively overcomes the disadvantages of the classical algorithms, including their precocious convergence and tendency to fall into the local optimum. The IEPSO shows an ideal global optimization performance and indicates a high application value for solving practical engineering optimization problems.

## Figures and Tables

**Table 1:** 11 test functions.

No.	Test function	*S*	CF
*f*_1_	Sphere: *f*_1_(*x*)=∑_*i*=1_^*D*^*x*_*i*_^2^ [10]	[−100,100]^*D*^	0
*f*_2_	Schaffer: $f\left(x,y\right)=0.5+{\left(\text{si}{\text{n}}^{2}\sqrt{x^{2}+y^{2}}-0.5\right)/\left(1+0.001\left(x^{2}+y^{2}\right)\right)}^{2}$ [33]	[−100,100]^*D*^	0
*f*_3_	Step: *f*_3_(*x*)=∑_*i*=1_^*D*^[*x*_*i*_+0.5]^2^ [10]	[−100,100]^*D*^	0
*f*_4_	SumSquares: *f*_4_(*x*)=∑_*i*=1_^*D*^*ix*_*i*_^2^ [10]	[−10,10]^*D*^	0
*f*_5_	Zakharov: *f*_5_(*x*)=∑_*i*=1_^*D*^*x*_*i*_^2^+(∑_*i*=1_^*D*^0.5×*ix*_*i*_^2^)^2^+(∑_*i*=1_^*D*^0.5×*ix*_*i*_)^4^ [10]	[−100,100]^*D*^	0

*f*_6_	Griewank: $f_{6}\left(x\right)=1/4000\sum_{i=1}^{D}x_{i}^{2}-\prod_{i=1}^{D}\cos\left(x_{i}/\sqrt{i}+1\right)$ [10]	[−600,600]^*D*^	0
*f*_7_	Rastrigin: *f*_7_(*x*)=∑_*i*=1_^*D*^[*x*_*i*_^2^ − 10cos(2*πx*_*i*_+10)] [10]	[−5.12,5.12]^*D*^	0
*f*_8_	Alpine: *f*_8_(*x*)=∑_*i*=1_^*D*^(|*x*_*i*_sin*x*_*i*_|+0.1*x*_*i*_) [6]	[−10,10]^*D*^	0
*f*_9_	Shubert: min*f*_9_(*x*, *y*)={∑_*i*_^5^*i*cos[(*i*+1)*x*+*i*]} × {∑_*i*_^5^*i*cos[(*i*+1)*y*+*i*]}	[−10,10]^*D*^	−186.731
*f*_10_	Ackley: $f_{10}\left(x\right)=-20\exp\left(-0.2\sqrt{1/D\sum_{i=1}^{D}x_{i}^{2}}\right)-\exp\left(1/D\sum_{i=1}^{D}\cos2\pi x_{i}\right)+20+e$ [10]	[−32,32]^*D*^	0
*f*_11_	Cmfun: $f_{11}\left(x,y\right)=x\sin\left(\sqrt{\left|x\right|}\right)+y\sin\left(\sqrt{\left|y\right|}\right)$	[−500,500]	−837.966

**Table 2:** Unimodal test functions.

Functions	Criteria	*C*_3_ = 2	*C*_3_ = 2∼0 *k* = −1	*C*_3_ = 2∼0 *k* = 1
*f*_1_	Mean	7.22*E* + 02	1.07*E* − 06	**4.50*E*** − **20**
	SD	3.97*E* + 04	1.11*E* − 12	**3.75*E*** − **16**
	Best	4.05*E* + 02	2.41*E* − 08	**1.55*E*** − **25**

*f*_2_	Mean	2.50*E* − 06	2.22*E* − 17	**0**
	SD	2.32*E* − 12	2.59*E* − 33	**0**
	Best	2.85*E* − 07	0	**0**

*f*_3_	Mean	1.99*E* + 02	8.03*E* − 07	**1.82*E*** − **20**
	SD	2.15*E* + 04	1.04*E* − 12	**1.05*E*** − **39**
	Best	35.81	3.95*E* − 08	**3.22*E*** − **24**

*f*_4_	Mean	7.110	2.45*E* − 08	**8.20*E*** − **20**
	SD	9.57	7.95*E* − 16	**5.11*E*** − **38**
	Best	1.47	4.08*E* − 09	**8.43*E*** − **26**

*f*_5_	Mean	1.74*E* + 03	3.86*E* − 04	**5.56*E*** − **11**
	SD	2.44*E* + 05	1.49*E* − 07	**4.88*E*** − **14**
	Best	8.29*E* + 02	9.78*E* − 06	**3.54*E*** − **11**

**Table 3:** Multimodal test functions.

Functions	Criteria	*C*_3_ = 2	*C*_3_ = 2∼0 *k* = −1	*C*_3_ = 2∼0 *k* = 1
*f*_6_	Mean	1.10	8.18*E* − 02	**4.92*E*** − **02**
	SD	4.6*E* − 03	8.37*E* − 04	**5.96*E*** − **04**
	Best	0.96	4.33*E* − 02	**1.23*E*** − **02**

*f*_7_	Mean	35.03	4.10	**1.9*Ee*** − **04**
	SD	8.44	2.5461	**5.649**
	Best	29.67	2.057	**2.25*E*** − **05**

*f*_8_	Mean	2.93	1.33*E* − 03	**5.28*E*** − **10**
	SD	0.30	3.10*E* − 08	**2.23*E*** − **12**
	Best	2.02	1.34*E* − 05	**5.83*E*** − **13**

*f*_9_	Mean	−186.7295	−**186.7309**	−**186.7309**
	SD	1.20*E* − 06	**0**	**0**
	Best	−186.7307	−**186.7309**	−**186.7309**

*f*_10_	Mean	7.649	2.35*E* − 04	**1.84*E*** − **11**
	SD	0.415	4.39*E* − 09	**2.27*E*** − **22**
	Best	6.513	5.73*E* − 05	**2.50*E*** − **12**

*f*_11_	Mean	−**837.9658**	−**837.9658**	−**837.9658**
	SD	**4.50*E*** − **09**	**0**	**4.40*E*** − **09**
	Best	−**837.9658**	−**837.9658**	−**837.9658**

**Table 4:** Unimodal test functions.

Functions	Criteria	*C*_3_ = 2∼0 *k* = 0.2	*C*_3_ = 2∼0 *k* = 2	*C*_3_ = 2∼0 *k* = 1
*f*_1_	Mean	**2.66*E*** − **20**	5.51*E* − 10	4.50*E* − 20
	SD	**2.65*E*** − **39**	2.87*E* − 19	3.75*E* − 16
	Best	**9.12*E*** − **24**	1.38*E* − 11	1.55*E* − 25

*f*_2_	Mean	**0**	**0**	**0**
	SD	**0**	**0**	**0**
	Best	**0**	**0**	**0**

*f*_3_	Mean	6.21*E* − 19	6.04*E* − 10	**1.82*E*** − **20**
	SD	2.63*E* − 36	7.79*E* − 19	**1.05*E*** − **39**
	Best	1.81*E* − 27	3.08*E* − 11	**3.22*E*** − **24**

*f*_4_	Mean	**1.70*E*** − **21**	2.42*E* − 11	8.20*E* − 20
	SD	**1.31*E*** − **41**	4.40*E* − 22	5.11*E* − 38
	Best	**2.82*E*** − **29**	4.36*E* − 12	8.43*E* − 26

*f*_5_	Mean	1.65*E* − 10	2.83*E* − 11	**5.56*E*** − **11**
	SD	3.30*E* − 20	3.59*E* − 11	**4.88*E*** − **14**
	Best	2.17*E* − 11	1.00*E* − 11	**3.54*E*** − **11**

**Table 5:** Multimodal test functions.

Functions	Criteria	*C*_3_ = 2∼0 *k* = 0.2	*C*_3_ = 2∼0 *k* = 2	*C*_3_ = 2∼0 *k* = 1
*f*_6_	Mean	**4.19*E*** − **02**	4.79*E* − 02	4.92*E* − 02
	SD	**3.43*E*** − **04**	7.07*E* − 04	5.96*E* − 04
	Best	**1.25*E*** − **02**	5.7*E* − 03	1.23*E* − 02

*f*_7_	Mean	4.46*E* − 03	**5.00*E*** − **05**	1.9*Ee* − 04
	SD	1.73*E* − 04	**3.03*E*** − **06**	5.649
	Best	2.31*E* − 12	**3.89*E*** − **11**	2.25*E* − 05

*f*_8_	Mean	**2.42*E*** − **10**	3.74*E* − 10	5.28*E* − 10
	SD	**6.74*E*** − **20**	2.47*E* − 12	2.23*E* − 12
	Best	**3.71*E*** − **16**	4.36*E* − 11	5.83*E* − 13

*f*_9_	Mean	−**186.7309**	−**186.7309**	−**186.7309**
	SD	**0**	**0**	**0**
	Best	−**186.7309**	−**186.7309**	−**186.7309**

*f*_10_	Mean	**1.13*E*** − **11**	2.05*E* − 10	1.84*E* − 11
	SD	**2.21*E*** − **22**	4.37*E* − 12	2.27*E* − 22
	Best	**5.06*E*** − **14**	1.75*E* − 10	2.50*E* − 12

*f*_11_	Mean	−**837.9658**	−**837.9658**	−**837.9658**
	SD	**0**	**0**	**4.40*E*** − **09**
	Best	−**837.9658**	−**837.9658**	−**837.9658**

**Table 6:** Parameter settings.

Algorithm	Population	Maximum iteration	Dim of each object	Others
PSO	40	1000	10	*C*_1_ = *C*_2_ = 2; *R*_1_ = *R*_2_ = 0.5
SPSO	40	1000	10	*ω* = 0.9–0.4; *C*_1_ = *C*_2_ = 2; *R*_1_ = *R*_2_ = 0.5
DE	40	1000	10	—
GA	40	1000	10	*GGAP = 0.5*; *PRECI = 25*
IEPSO	40	1000	10	*ω* = 0.9–0.4; *C*_1_ = *C*_2_ = 2; *C*_3_ = 2–0; *R*_1_ = *R*_2_ = *R*_3_ = 0.5

**Table 7:** Unimodal test functions.

Functions	Criteria	PSO	SPSO	DE	IEPSO	GA
*f*_1_	Mean	1.33*E* + 03	3.08*E* + 03	7.31*E* − 12	**8.92*E*** − **22**	11.696
	SD	2.53*E* + 05	1.21*E* + 06	2.25*E* − 23	**2.65*E*** − **39**	44.192
	Best	1.14*E* + 03	1.20*E* + 03	2.42*E* − 12	**7.72*E*** − **27**	4.660

*f*_2_	Mean	2.96*E* − 02	8.80*E* − 02	8.37*E* − 06	**0**	1.79*E* − 11
	SD	8.36*E* − 04	8.96*E* − 04	1.58*E* − 10	**0**	0
	Best	4.55*E* − 03	8.428734	7.55*E* − 10	**0**	1.79*E* − 11

*f*_3_	Mean	1.19*E* + 03	2.51*E* + 03	1.14*E* − 11	**6.21*E*** − **19**	7.430
	SD	2.93*E* + 05	1.82*E* + 06	9.95*E* − 23	**2.63E** − **36**	5.833
	Best	1.06*E* + 03	2.82*E* − 02	2.10*E* − 12	**1.81*E*** − **27**	4.542

*f*_4_	Mean	82.38	82.10	3.36*E* − 13	**1.70*E*** − **21**	3.031
	SD	6.86*E* + 02	1.40*E* + 03	9.95*E* − 26	**1.31*E*** − **41**	0.835
	Best	1.15*E* + 02	37.39	1.15*E* − 13	**2.82*E*** − **29**	1.968

*f*_5_	Mean	1.26*E* + 04	8.60*E* + 03	**7.02*E*** − **12**	1.65*E* − 10	3.62*E* + 03
	SD	2.06*E* + 07	2.15*E* + 07	**1.81*E*** − **23**	3.30*E* − 20	3.44*E* + 05
	Best	1.04*E* + 04	1.30*E* + 02	**2.67*E*** − **12**	2.17*E* − 11	2.53*E* + 03

**Table 8:** Multimodal test functions.

Functions	Criteria	PSO	SPSO	DE	IEPSO	GA
*f*_6_	Mean	1.548	1.752	9.44*E* − 02	**4.19*E*** − **02**	1.006
	SD	0.026	0.093	4.87*E* − 04	**3.43*E*** − **04**	0.018
	Best	1.236	1.417	0.06	**0.013**	0.794

*f*_7_	Mean	57.737	43.405	11.945	**4.46*E*** − **03**	8.939
	SD	117.768	65.178	16.502	**1.73*E*** − **04**	3.608
	Best	35.981	3.17*E* + 01	6.398	**2.31*E*** − **12**	5.040

*f*_8_	Mean	4.996	4.665	3.79*E* − 02	**2.42*E*** − **10**	0.423
	SD	1.91*E* + 00	1.056	5.4*E* − 03	**6.74*E*** − **20**	0.051
	Best	2.933	3.151	4.6*E* − 03	**3.71*E*** − **16**	0.086

*f*_9_	Mean	−186.448	−186.048	−186.728	−**186.731**	−**186.731**
	SD	1.19*E* − 01	9.83*E* − 01	2.29*E* − 08	**0**	**9.99*E*** − **12**
	Best	−1.87*E* + 02	−186.731	−186.7309	−**186.7309**	−**186.731**

*f*_10_	Mean	13.134	15.560	1.613	**1.13*E*** − **11**	2.515
	SD	14.260	2.163	0	**2.21*E*** − **22**	0.166
	Best	2.861	12.719	1.613	**5.06*E*** − **14**	1.796

*f*_11_	Mean	−740.326	−715.438	−**837.966**	−**837.966**	−**837.966**
	SD	8.74*E* + 03	7.23*E* + 03	**0**	**0**	**0**
	Best	−837.966	−837.697	−**837.966**	−**837.966**	−**837.966**

**Table 9:** Three improved particle swarm algorithm test results.

Functions	Criteria	IEPSO	DMSDL-PSO [25]	BHPSOWM [26]
*f*_1_	Mean	**8.92*E*** − **22**	4.73*E* − 10	42.40
	SD	**2.65*E*** − **39**	1.81*E* − 09	52.11

*f*_3_	Mean	**6.21*E*** − **19**	2.37*E* + 03	7.61
	SD	**2.63*E*** − **36**	5.71*E* + 02	0.07

*f*_6_	Mean	4.19*E* − 02	**8.66*E*** − **05**	—
	SD	3.43*E* − 04	**2.96*E*** − **04**	—

*f*_7_	Mean	**4.46*E*** − **03**	9.15*E* + 01	76.18
	SD	**1.73*E*** − **04**	1.80*E* + 01	26.75

*f*_8_	Mean	**2.42*E*** − **10**	1.31*E* + 02	—
	SD	**6.74*E*** − **20**	5.82*E* + 01	—

*f*_10_	Mean	**1.13*E*** − **11**	1.01*E* + 00	1.72
	SD	**2.21*E*** − **22**	2.71*E* − 01	0

## Data Availability

The data used to support the findings of this study are available from the corresponding author upon request.
